# Corneal Confocal Microscopy and the Nervous System: Introduction to the Special Issue

**DOI:** 10.3390/jcm11061475

**Published:** 2022-03-08

**Authors:** Rayaz A. Malik, Nathan Efron

**Affiliations:** 1 Weill Cornell Medicine-Qatar, Research Division, Qatar Foundation, Education City, Doha 24144, Qatar; ram2045@qatar-med.cornell.edu; 2 School of Optometry and Vision Science, Queensland University of Technology, Kelvin Grove, QLD 4059, Australia

## 1. Introduction

The heretical idea that corneal confocal microscopy (CCM)—an ophthalmic instrument—could be used to assess neurological disease emerged around the turn of the 21st century. CCM allows the corneal ultrastructure to be examined in vivo at 700× magnification; the standard clinical instrument used for examining the cornea—the slip-lamp biomicroscope—only has a maximum magnification of 40×. Thus, CCM has enabled the discovery of previously unknown features of the cornea and the observation of known features at much higher magnifications and resolutions—a true revolution in ophthalmic science.

The repurposing of CCM to study diabetic neuropathy in the first instance, and, subsequently, a broader array of neurological disorders, is a wonderful illustration of challenging dogma in medicine.

We are delighted to write this cover editorial, which provides an overview of the past, present and future of CCM as a tool for investigating the nervous system. This editorial is unapologetically somewhat of a personal account, given our deep involvement in the development of this application from almost the beginning.

## 2. Discovering CCM

The optical principles of confocal microscopy were described by Minsky in 1955, who cited his rationale for developing this device “to better understand the interconnection of nerve cells” [1]. The first functional microscope was developed by Petran et al. [2] in 1960, but it was not until 1988 that Dilly [3] demonstrated how the device developed by Petran et al. could be mounted horizontally and used to examine the human cornea in vivo. The first scans were captured from Dilly’s own eye [3].

As an academic optometrist with a research interest in the impact of contact lenses on the cornea, I (N.E.) first learned about CCM at a contact lens conference in Hawaii, USA, in August 1988. I sat in on a lecture by ophthalmologist Dwight Cavanagh, who had very recently developed his own CCM. Coincidentally, Dilly’s seminal CCM paper [3] was published during the same week as this conference, but I had not seen it.

Renowned for his often flowery and effusive oratory, Cavanagh stepped up to the podium and commenced his lecture with a profound statement that stunned the audience: “It’s very rare in one’s life to ever be a part of something that’s really fundamental in science that changes the way everything is done. I guess there are points like that in the history of science, when Isaac Newton saw the apple fall off the tree, when Galileo Galilei looked through his first telescope, and when Antonie van Leeuwenhoek found the little animals in pond water; but believe me … confocal microscopy is one of those branch points in science, not just cell biology or ophthalmology or optometry or contact lenses. It is a new paradigm, a … microscope that lets us see things nobody else has ever been able to see before” [4].

Cavanagh proceeded to show images of the cornea obtained using CCM, which ‘blew me away’. I (N.E.) immediately saw the potential application of CCM to help understand the ocular response to contact lens wear. At great expense, I managed to obtain one of the first commercially available CCM instruments—the Tomey ConfoScan Confocal Microscope Model P4 (Tomey, Erlangen, Germany)—soon after it came onto the market in 1996.

I first spent a few weeks scanning the corneas of fellow researchers. One feature of corneal anatomy in particular that caught my attention was the sub-basal nerve plexus. This plexus was known to exist from ex vivo histological studies but had never before been seen in the living human eye. Recognizing the importance of this anatomical feature, I conducted a study with graduate optometry student Laura Oliveira-Soto, describing the morphology of the sub-basal nerve plexus in great detail [5].

## 3. Translating CCM from Ophthalmology to Neurology

No sooner had our paper describing the corneal sub-basal nerve plexus been published, on 1 May 2001 [5], than my annual review was due with my diabetes doctor, who happened to be Professor Andrew Boulton at the Manchester Royal Infirmary. Having gained weight and been less than attentive with my glycaemic control, more so to distract him, I handed him a copy of this paper [5]. At the time, I was unaware that Andrew was a noted authority on diabetic peripheral neuropathy (I had never Googled him to find out his academic interests). Naturally, therefore, Andrew was very interested, but he was time-poor; so, on 14 June 2001, he wrote me a letter stating: “Turning now to your controcal [*sic*] microscope … Rayaz [Malik] and I could possibly visit your Centre … and think of some collaborative work in diabetic neuropathy” [6].

When I met R.M., he was skeptical at first (“these are 5th cranial nerves, but I am more interested in small peripheral nerves”); nevertheless, he suggested we use CCM to assess a small cohort of diabetic patients. Recruitment was initially slow, as R.M. relied on patients in his clinic making their own way across the city to UMIST to undergo CCM. However, the study was rapidly completed when he offered to drive them personally to UMIST in his Porsche! This was the beginning of an extremely fruitful collaboration which enabled us to effectively translate CCM from the world of ophthalmology/optometry to neurology. Thus, I (N.E.) found myself to be in the right place, at the right time, working with the right people, and asking the right questions.

As an aside, I (N.E.) have continued with contact lens research alongside my diabetes/neurology research; CCM has also turned out to be a valuable tool for understanding the response of the cornea to contact lens wear [7], which was my original reason for obtaining a CCM.

## 4. According Precedence

The paper by Laura Oliveira-Soto and N.E. describing the detailed anatomy of corneal sub-basal nerves [5] was submitted to the journal *Cornea* in September 2000. In this paper, we concluded, “This study provides convincing evidence of the suitability of confocal microscopy to image corneal nerves”, and “Future studies should investigate the … morphology and architecture of corneal nerves in … diabetic patients …” [5].

In October 2000, Rosenberg et al. published a paper [8] which used a tandem scanning confocal microscope to show a progressive loss of long nerve fibre bundles with increasing severity of diabetic neuropathy; they concluded “Confocal microscopy appears to allow early detection of beginning neuropathy” [8]. In science, precedence must be accorded to those who publish first, so without doubt, credit must go to Rosenberg et al. [8] for being the first to describe corneal nerve loss in diabetic peripheral neuropathy.

We (R.M. and N.E.) had commenced our study in patients with diabetes in August 2001 and applied rigorous morphometric techniques, which R.M. had learnt from quantifying myelinated and unmyelinated nerve fibers in sural nerve biopsies from patients with diabetic neuropathy [9], to quantify corneal nerve fiber morphology. Our paper [10], which was submitted to *Diabetologia* in November 2002 and published in May 2003, rather ambitiously concluded “Corneal confocal microscopy is a rapid, non-invasive in vivo clinical examination technique which accurately defines the extent of corneal nerve damage and repair and acts as a surrogate measure of somatic neuropathy in diabetic patients. It could represent an advance to define the severity of neuropathy and expedite assessment of therapeutic efficacy in clinical trials of human diabetic neuropathy” [10].

## 5. CCM in Diabetic Neuropathy

We initially secured an innovation grant from the JDRF to build on our pilot data to show that CCM could identify diabetic patients with sub-clinical neuropathy and progressive worsening with increasing severity of diabetic neuropathy. Given the reluctance/hostility amongst some in the neurology community to accept that an ophthalmic instrument quantifying ‘short cranial nerves’ could reflect a dying back neuropathy affecting ‘long sensory nerves’, we knew we had to secure further financial support to increase the size of the cohorts, establish longitudinal change, and demonstrate response to interventions.

We were fortunate to secure an RO1 grant from the NIDDK and a substantial grant from the JDRF to conduct the LANDMARK study (Longitudinal Assessment of Neuropathy in Diabetes using novel ophthalmic MARKers) [11]. This study generated valuable cross-sectional and longitudinal CCM, neurological and general medical data on 231 type 1 and 2 diabetic patients with and without neuropathy, and 61 non-diabetic subjects, all of which served as a valuable contemporary control and historic reference set for future work.

Subsequent funding from the NIDDK enabled the establishment of an international consortium, which combined data from ~1000 patients with type 1 and type 2 diabetes and impaired glucose tolerance and showed that CCM has excellent diagnostic [12,13] and prognostic [14] value and could detect early nerve regeneration after simultaneous pancreas and kidney transplantation in patients with type 1 diabetes [15,16]. Age-adjusted normative values for CCM were established [17], and we showed that the severity of corneal nerve loss and associated risk factors are different for type 1 and type 2 diabetic patients [18].

Corneal nerve loss is found in children [19,20] and adults [21] with type 1 diabetes, prior to developing retinopathy and microalbuminuria, and in patients with impaired glucose tolerance [22,23] and type 2 diabetes that has been recently diagnosed [24]. Painful diabetic neuropathy is associated with greater corneal nerve loss at the inferior whorl [25] and is related to pain severity [26]. Corneal nerve loss is evident in patients with diabetic autonomic neuropathy [27,28,29,30,31] and in men with type 1 and type 2 diabetes and erectile dysfunction [32,33]. Reduced corneal nerve fibre length (CNFL) predicts 4-year incident diabetic peripheral neuropathy [34,35]. A more rapid decline in CNFL has been associated with the development of diabetic peripheral neuropathy [36] and Charcot foot with foot ulceration [37].

## 6. CCM in Synergy with Other Ophthalmic Markers of Diabetic Neuropathy

Our work with the CCM led us to develop a more comprehensive neurological model incorporating other ophthalmic markers of diabetic peripheral neuropathy [38]. We demonstrated a commensurate loss of corneal sensitivity [39]. We also hypothesised that, given that the fifth cranial nerve is impacted, other ocular cranial nerves, such as the second (optic) cranial nerve, could also be involved. Our subsequent investigations using the newly developed technique of optical coherence tomography demonstrated significant thinning of the retinal nerve fibre layer in patients with diabetic peripheral neuropathy [40]. We also found a commensurate loss in visual sensitivity with the central 30° of the visual field in these patients [41].

## 7. CCM in Other Peripheral Neuropathies

Chemotherapy-induced peripheral neuropathy is characterised by pain and reduced quality of life and can lead to dose reduction or discontinuation from chemotherapy. Corneal nerve loss is observed in patients with various forms of cancer, and recovery has been demonstrated following chemotherapy [42,43,44].

Stettner et al. [45] showed significant corneal nerve loss and an increase in dendritic cells in patients with chronic inflammatory demyelinating neuropathy, multifocal motor neuropathy, and monoclonal gammopathy of unknown significance, which were associated with disease severity and pain. Increased corneal inflammatory cells predict disease progression with high sensitivity [46,47]. Corneal nerve loss occurs in patients with HIV-associated neuropathy [48]. Corneal nerve fractal dimension may differentiate HIV neuropathy from diabetic neuropathy [49]. Idiopathic small fibre neuropathy [50] is associated with significant corneal nerve loss [51], with an increased detection rate for this condition using CCM [52].

Corneal nerve loss occurs in patients with Charcot Marie Tooth disease type 1A [53] and in severe peripheral neuropathy associated with a rare nerve growth factor-β mutation [54]. We have also demonstrated corneal nerve loss in patients with Friedreich’s ataxia [55] and neurofibromatosis type 1 [56].

The fatal inherited disorder, transthyretin familial amyloid polyneuropathy, is characterized by a progressive neuropathy and cardiomyopathy [57]; CNFL is reduced in patients with this condition [58] and has good diagnostic utility [59].

Patients with Fabry’s disease demonstrate small fibre neuropathy as a result of nerve damage induced by globotriaosylceramide (GI_3_) [60]. Corneal nerve loss is demonstrated in patients with Fabry disease [61], which is correlated with disease severity [62]. Corneal nerve fibre density (CNFD) was reduced in patients with hypothyroidism but improved after levo-thyroxine treatment [63]. In patients with fibromyalgia, stromal nerve thinning reduced sub-basal nerve fibre density and was related to a variety of pain descriptors [64,65,66,67].

## 8. CCM in Central Neurodegenerative Disease

A number of studies have recently investigated whether corneal nerve loss could serve as a surrogate marker of neurodegeneration in central neurodegenerative diseases. Parkinson’s disease (PD) is associated with reduced corneal sensitivity and compromised corneal nerve morphology [68,69,70].

In patients with PD, loss of corneal nerves is associated with the severity of cognitive dysfunction [71] and with altered trigeminal nerve white matter diffusion properties [72]. Altered corneal nerve morphology has been shown in patients with PD [73]. We have confirmed corneal nerve fibre loss in PD [74], with CNFD demonstrating excellent diagnostic utility [75]. A lower CNFL predicts progressive worsening of UPDRS-III over 12 months in patients with PD [76], suggesting the application of CCM in diagnosing pre-motor PD.

Four recent studies [77,78,79] have demonstrated reduced sub-basal corneal nerve density in patients with multiple sclerosis. Corneal nerve loss is also associated with the bulbar function disability score in patients with amyotrophic lateral sclerosis [80] and with disease severity and progression [81].

Corneal nerve loss occurs in patients with transient ischaemic attack and minor stroke [82], major stroke [83], and recurrent stroke [84] and is associated with the presence of white matter hyperintensities [85]. In patients with mild cognitive impairment and dementia, corneal nerve fibre loss is associated with the degree of cognitive impairment and physical disability [86,87]. The severity of cerebral ischemia is associated with cognitive impairment, brain atrophy, and corneal nerve loss in mild cognitive impairment and dementia [88].

Corneal nerve fibre density and length are reduced in patients with migraine [89], trigeminal neuralgia [90,91], and burning mouth syndrome [92].

## 9. CCM in Clinical Trials

Corneal nerve fibers have been shown to regenerate 6 months after simultaneous pancreas and kidney (SPK) transplantation in patients with type 1 diabetes [93]. We have also shown corneal nerve regeneration 24 months after an improvement in glycemia, blood pressure, and lipids [94] and 12 months after SPK transplantation; there were no changes in symptoms, neurophysiology, quantitative sensory testing, or skin biopsy results [15]. We have more recently shown increased CNFL 12 months after SPK transplantation followed by improved neuropathic symptoms after 24 months and neurophysiology after 36 months [16].

We have demonstrated with CCM that there is a recovery of small-fiber neuropathy in type 1 diabetes subjects using continuous subcutaneous insulin infusion compared with those using multiple daily injection [95].

Bariatric surgery improves in corneal nerve morphology in obese subjects with [96] and without [97] diabetes. A new anti-inflammatory peptide, ARA290-Cibinetide, improves corneal nerve morphology in patients with sarcoidosis-related neuropathy [98,99] and type 2 diabetes [100], with commensurate improvement in pain scores. Improvement in corneal nerve morphology strongly correlates with the GAP-43^+^ expression in skin biopsies, indicating intraepidermal nerve fibre repair and with an improvement in pain intensity after 28 days [99].

Seal oil omega-3 polyunsaturated fatty acid increased CNFL, with no change in nerve conduction velocity and sensory function over 12 months, in patients with type 1 diabetes [101]. Omega-3 fatty acid was also found to increase CNFL in patients with type 1 diabetes after 180 days, without change in thermal thresholds, autonomic function, or nerve conduction studies [102].

In a 12-month study of weekly GLP-1 or basal bolus insulin, corneal nerve regeneration occurred, but there was no change in vibration perception or sudomotor function [103].

## 10. Future Developments of CCM

Several slit scanning in vivo CCMs are commercially available, including Tomey Corporation (Cambridge, MA, USA), Nidek Technologies (Gamagori, Japan) and Helmut Hund (Wetzlar, Germany), but they have limited image resolution for the sub-basal nerve plexus. The laser-scanning CCM (Heidelberg Retina Tomograph III Rostock Corneal Module, Heidelberg Engineering GmbH, Heidelberg, Germany) is capable of generating very high resolution and high contrast images of sub-basal nerves. Looking to the future, a non-contact CCM would make image acquisition easier and facilitate widespread clinical application; a non-contact attachment for the Heidelberg instrument has been developed [104], but image acquisition of the corneal sub-basal nerve plexus is very difficult [104].

A limitation of CCM is its small field of view, and some centers have used wide-field imaging to create maps of the sub-basal nerve plexus [105,106,107]. The main morphological parameters quantified include corneal nerve fiber density, branch density, fiber length, and inferior whorl length [108,109]. Corneal nerve fractal dimension analysis [49] may help to differentiate neuropathies of differing aetiology [110]. To expedite unbiased corneal nerve quantification, CCMetrics and ACCMetrics are freely available software for manual, semi-automated, and fully automated quantification of sub-basal corneal nerves [111,112]. Novel artificial-intelligence-based algorithms have also been developed for fully automated corneal nerve quantification [113] and disease severity classification in diabetic neuropathy [114].

## 11. Bibliometrics of CCM Research for Assessing Corneal Nerves in Neuropathy

There has been an almost exponential growth of research into use of CCM to assess corneal nerves in patients with various forms of ophthalmic and systemic neuropathy. To illustrate this, we searched the Scopus database on 1 March 2022, for papers published since 2000, using the following search term:

TITLE-ABS-KEY(cornea*) AND TITLE-ABS-KEY(“confocal microscop*”) AND TITLE-ABS-KEY(nerv* OR neura* OR neuro*) AND (PUBYEAR > 1999) AND (LIMIT-TO(LANGUAGE, “English”)) AND (LIMIT-TO(SRCTYPE, “j”))

A total of 1343 papers were captured by this search. These papers have collectively attracted 37,304 citations and have been published in >160 journals by >160 authors from >160 institutions (Scopus caps counts at 160) in 63 countries. Only 10 papers were published in year 2000 versus 179 in 2021 (i.e., more than 3 papers per week in 2021).

Although CCM was originally intended for ophthalmic research, the majority of papers captured appear to relate to systemic rather than ocular neuropathy. The five most prolific authors, who all happen to work primarily in the field of systemic neuropathy, are R.M. (163 papers), Ioannis Petropoulos (77), N.E. (74), George Ponirakis (62), and Maryam Ferdousi (53). The field as defined by the above search term has a h-index of 90.
